# Correlation between increased serum malondialdehyde and spectrum of cranial ultrasonography findings in hypoxic ischemic encephalopathy: could it be used as a predictor of disease severity?

**DOI:** 10.1186/s43055-020-00369-x

**Published:** 2020-12-05

**Authors:** Hadeel M. Seif El Dein, Nouran Fahmy, Zahraa Ezz El Din, Marianne Morgan, Marwa Abdel Fattah, Sara S. Eltatawy

**Affiliations:** 1 Department of Radiology, Cairo University, Cairo, Egypt; 2 Department of Pediatrics, Cairo University, Cairo, Egypt; 3 Department of Chemical Pathology, Cairo University, Cairo, Egypt

**Keywords:** Hypoxic ischemic encephalopathy (HIE), Serum malondialdehyde (MDH), Cranial ultrasound

## Abstract

**Background:**

Hypoxic ischemic encephalopathy (HIE) is a major cause of mortality and morbidity in neonates. Malondialdehyde (MDH) is a colorless lipid that can be used as a marker for oxidative stress. Cranial ultrasound sensitivity and specificity in detection of neonatal HIE ought to be further investigated. This study aims to detect whether serum (MDH) can be used as an indicator for HIE severity and to assess the role of cranial ultrasound in diagnosis of HIE neurological disorders, correlating ultrasound findings to MDA levels.

**Results:**

Statistically significant differences were found between the serum MDA levels in patients compared to controls as well as among serum MDA in patients with advancing Sarnat stages (I, II, III) *P* value < 0.001. Statistically significant levels of serum MDA were found in patients with ischemic US findings compared to those with normal scan; 36.4% of cases with ischemic US findings were diagnosed as Sarnat stage II while 63.6% were diagnosed with stage III with a statistically significant difference (*P* = 0.016).

**Conclusion:**

Cranial ultrasound can be used for diagnosis of neonatal hypoxic ischemic insults, with lower sensitivity in mild cases and increased sensitivity in severe cases; and when combined with measuring serum MDA levels, it can be used as a diagnostic marker and as a predictor for severity of HIE.

## Background

Hypoxic ischemic encephalopathy (HIE) remains one of the major causes of mortality and morbidity in neonates [1]. Having a reliable biomarker to diagnose and predict the severity of this condition is a valuable tool in the clinical setting. Malondialdehyde (MDA) is a colorless lipid resulting from the effect of free oxygen radicals on tissues and from a series of reactions during lipid peroxidation [2]. Serum MDA concentrations vary with varying severity of HIE and could therefore be used as a predictor for determining HIE staging, treatment and prognosis [3]. Cranial ultrasound is an easily acquired bed-side, radiation-free examination; its sensitivity and specificity in detection of neonatal HIE needs to be further investigated [4].

The aim of our study was to detect the role of serum malondialdehyde (MDA) levels in HIE and to determine whether MDA can be used as an indicator of severity in HIE. We also aimed to detect MDA levels in correlation to cranial ultrasound findings in HIE patients.

## Methods

This prospective study was carried out in the Neonatal Intensive Care Unit (NICU) Maternal Hospital. Patient collection spanned over a period of 2 years starting September 2014. It included 2 groups; a study group (*n* = 42) and a control group (*n* = 42) adding up to a total of 84 subjects. The study group included 42 full-term and near-term (35–37 weeks) neonates who fulfilled the eligibility criteria for hypoxic ischemic encephalopathy (HIE). The control group, collected from the delivery room included 42 healthy neonates, matched for gestational age, gender, and birth weight.

Eligibility criteria for the stuFdy (hypoxic) group included near or full-term newborns delivered with indicators of peripartum hypoxia-ischemia and moderate to severe encephalopathy. Hypoxia-ischemia was defined as patients with Apgar scores less than 3 at 1 min and 5 at 5 min of life, arterial pH of ≤ 7.2, base deficit >− 10 mmol within 60 min of birth, and delayed first cry beyond 1 min of birth [5]. Encephalopathy was defined according to Sarnat criteria (lethargy, stupor, coma, abnormal tone, and/or seizures) [6]. The diagnosis of hypoxic ischemic encephalopathy was made by Neonatal ICU staff and attending physicians.

Infants were excluded from the study group if they were less than 35 weeks gestational age, had major congenital malformations, did not show the required evidence of hypoxia-ischemia at birth, or presented with hyperbilirubinemia.

The study was approved by local ethical committee and informed parental consent was given by all the patients in both groups. The following data were collected by NICU staff; perinatal history, gestational age using new Ballard score [7], birth weight, gender, mode of delivery, 1- and 5-min Apgar scores, and time of first cry. All patients were subjected to assessment of serum MDA within 24 h of life; the normal value of MDA using ELISA technique was defined as levels from 0.2 up to 1 nmol/ml [8].

Patients in the study hypoxic group were subjected to additional investigations including arterial blood for pH, complete blood picture (CBC), kidney function tests, liver function tests, coagulation profile, serum electrolytes, calcium levels, and random blood sugar. They underwent a clinical grading of neurological affection according to Sarnat and Sarnat Score after birth [6]. Other data such as presence of multisystem affection and need for inotropes was also recorded.

Due to early mortality, cranial ultrasound was only performed on 37/42 hypoxic group patients during their first week of life. This was done using the US machine TOSHIBA with multifrequency probe (5–10 MHz) through the anterior fontanel. Ischemic ultrasound findings were then correlated to MDA levels and clinical factors. Hypoxic ischemic affection was defined by ultrasound when the following findings were seen: brain edema with echogenic subcortical white matter, increased cerebral echogenicity with or without loss of gray-white matter differentiation, intraventricular bleeding, and/or thalamic or basal ganglia involvement [9]. Head sonography was also used to assess pulsed Doppler flow velocities and the resistive index of the cerebral arteries [10].

### Statistical methods

Data were analyzed using IBM© SPSS© Statistics version 23 (IBM© Corp., Armonk, NY, USA), MedCalc© version 15 (MedCalc© Software bvba, Ostend, Belgium). Normality of numerical data distribution was examined using the D’Agostino-Pearson test.

Discrete numerical variables were presented as median and interquartile range and intergroup differences were compared using the Mann-Whitney test.

Continuous numerical variables were presented as mean ± SD and intergroup differences were compared using the unpaired *t* test (for two-group comparison) or one-way analysis of variance (ANOVA) (for multiple-group comparison). The Tukey-Kramer post hoc test was used for pairwise comparison whenever the ANOVA test revealed a statistically significant difference among the groups.

Categorical variables were presented as number or proportion and percentage, and intergroup differences were compared using Fisher’s exact test.

Receiver-operating characteristic (ROC) curve analysis was used to examine the predictive value of serum MDA. The area under the various ROC curves (AUC) was compared with that of random prediction using the DeLong method.

Correlations among numerical variables were tested using the Spearman rank correlation. Time to event analysis was done using the Kaplan-Meier method, and the log-rank test was used to compare individual Kaplan-Meier curves.

A two-sided *P* value < 0.05 was considered statistically significant.

Results

In our study of 84 neonates, female to male ratio of the hypoxic group were 36% and 64%, respectively, while in the control group, gender was divided equally at 50%. Mean gestational age of hypoxic and control groups was 38.2 ± 1.4, and 38.0 ± 0.9, respectively. Mean birth weight of hypoxic and control groups was 3.07 ± 0.64 and 3.09 ± 0.4, respectively. Mode of delivery in the hypoxic group was 57% CS and 43 % vaginal delivery, while the control group was born 59.5% by CS and 40.5% by vaginal delivery.

Among maternal risk factors in our hypoxic group, hypertension proved to be the highest percentage at 33% followed by antepartum hemorrhage at 24%, although neither showed a statistically significant difference.

In terms of hypoxic events surrounding the birth, perinatal hypoxia showed a highly significant difference of Apgar scores at 1 and 5 min between hypoxic group and control group (*P* < 0.001).

As for encephalopathy, the most common neurological manifestations in the hypoxic group were seizures (88%) followed by hypotonia (74%), hyporeflexia (55%), disturbed conscious level (52%) irritability (24%), hyperreflexia (26%) and hypertonia (2%), which translated to 23% of hypoxic babies displaying Sarnat stages 1 and 2 and 52% displaying Sarnat stage 3

Multisystem affection is common in HIE babies; in our study, renal dysfunction was most frequent (99%) followed by metabolic acidosis (91%), pulmonary hypertension (60%), hypotension (52%), poor perfusion (48%), MAS (41%), bleeding tendency (38%), and hypoglycemia (10%). The most common complication in the hypoxic group was sepsis (43%) followed by pneumonia (12%). Outcome was unfavorable with a 43% mortality rate.

**Fig. 1:**
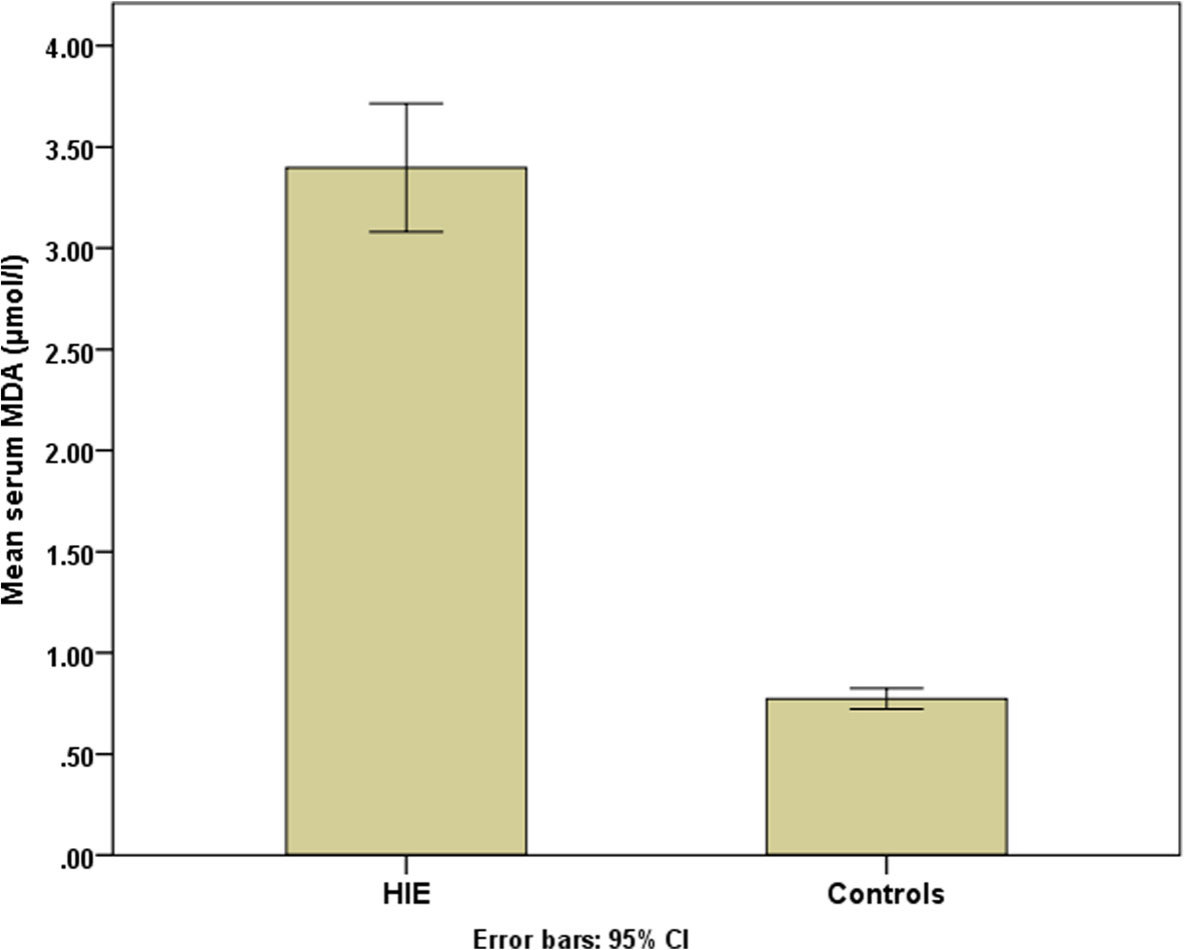
Mean serum (MDA) levels in hypoxic neonates and control groups

**Fig. 2:**
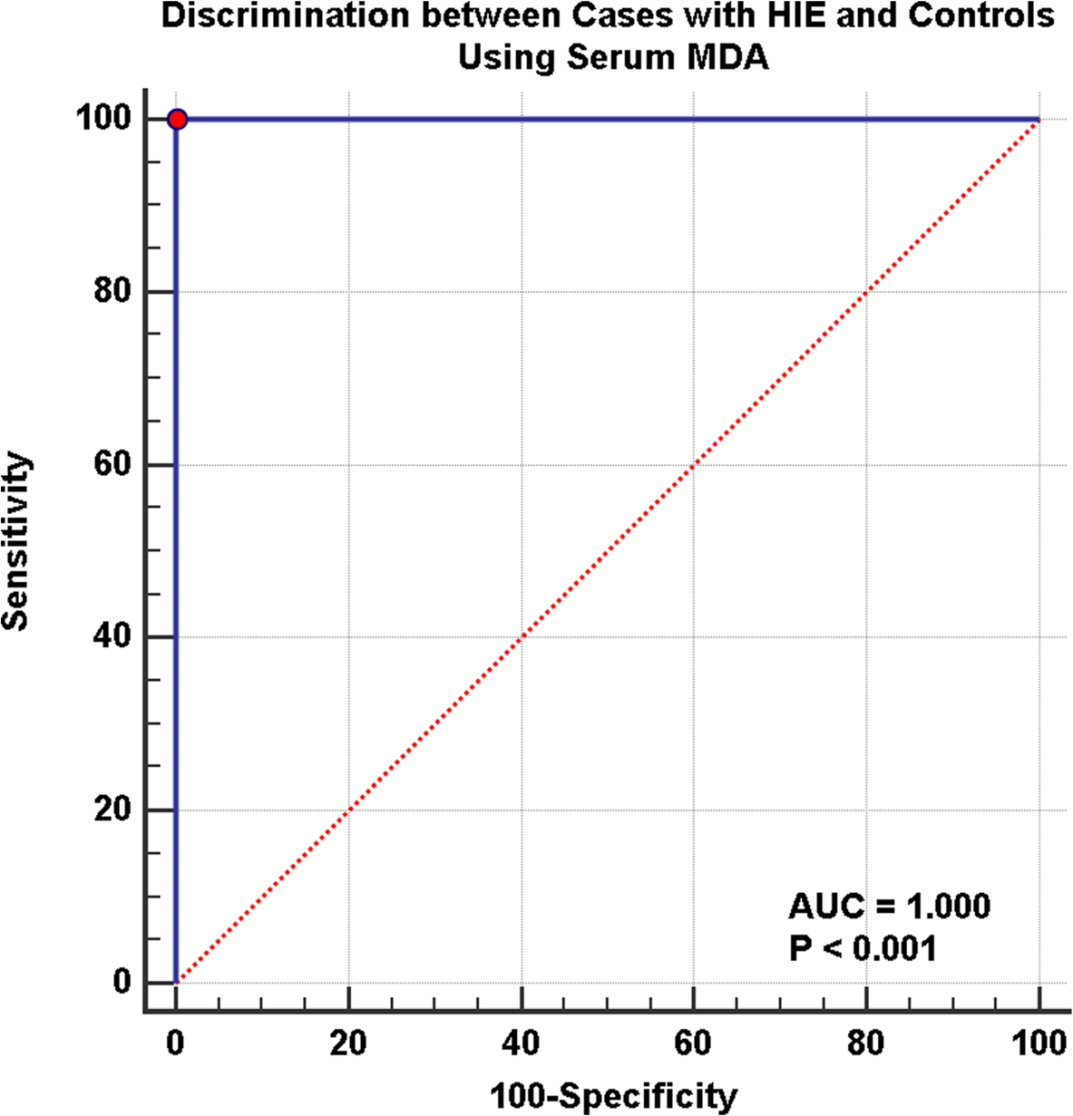
ROC curve for discrimination between neonates with HIE and controls using serum (MDA) levels

Our aim was to discover the role of MDA in HIE and correlate serum levels with severity of HIE. We found a highly significant difference between MDA levels in hypoxic neonates (3.40 ± 1.02) nmol/ml compared to the control group (0.77 ± 0.17) nmol/ml (*P* < 0.001) (Fig. 1) with a ROC curve showing MDA level sensitivity and specificity of 100% in hypoxic neonates compared to control group. Area under curve was 1 with highly significant difference (*P* < 0.001) (Fig. 2).

**Fig. 3:**
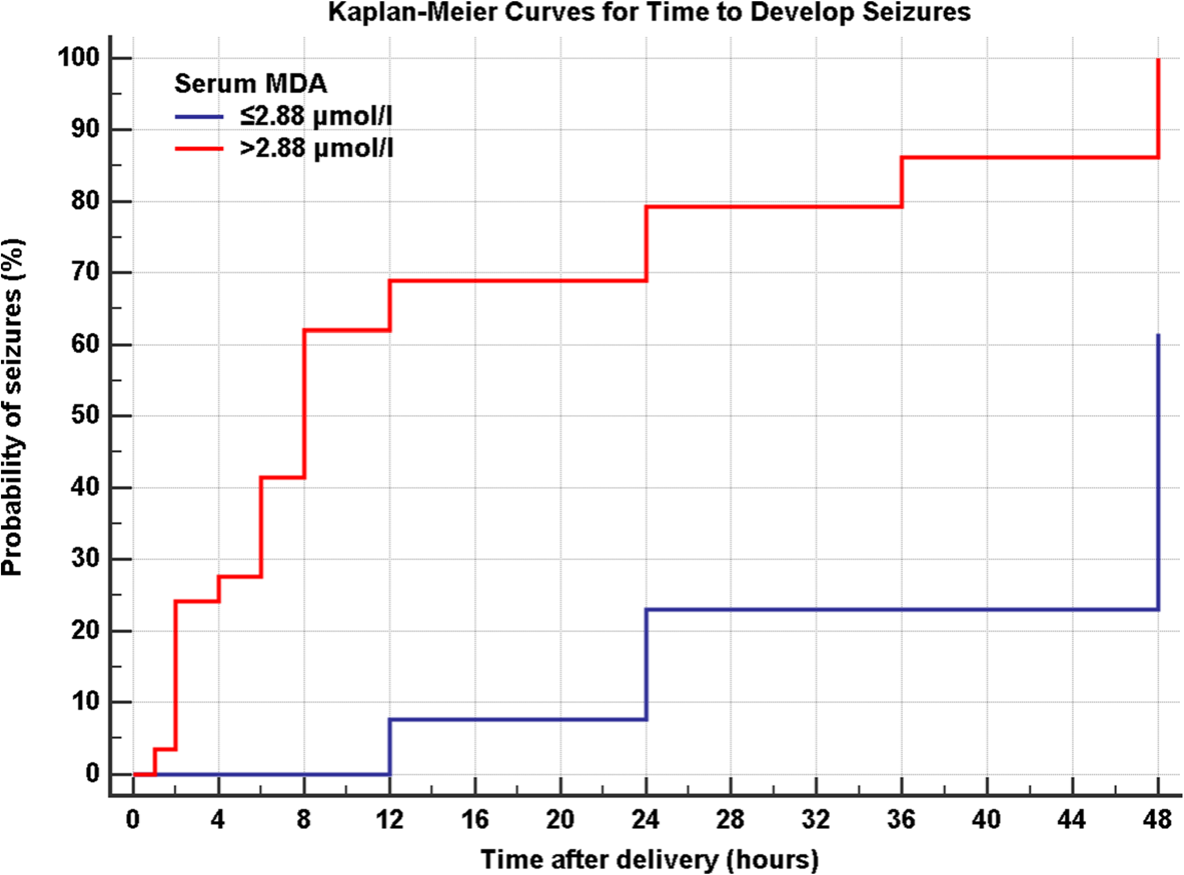
Kaplan-Meier curves for time to develop seizures in patients with serum MDA ≤ 2.88 nmol/ml or > 2.88 nmol/ml

As for severity of HIE, higher MDA levels correlated to presence and earlier onset of seizures in the hypoxic group (Table 1). A cut-off level of 2.4 nmol/ml for MDA showed 84% sensitivity and 100% specificity for occurrence of seizures. Concerning timing of seizures in hypoxic babies, our data shows that at a level of ≤ 2.88 nmol/ml, probability of occurrence of seizures before 12 h of life is extremely low (Fig. 3).

**Table 1:** Relationship between MDA and occurrence of seizures

Variable	Seizures (*n* = 37)	No seizures (*n* = 5)	T	df	*P* value¶
Serum MDA(nmol/ml)	3.57 ± 0.96	2.12 ± 0.18	8.166	35.662	**< 0.001**

Data are mean ± SD

¶Unpaired *t* Test

*t* T statistic, *df* Degree of freedom

Mean MDA levels were 2.16 ± 0.31 nmol/ml, 3.02 ± 0.67† nmol/ml, and 4.13 ± 0.66†‡ nmol/ml correlating to Sarnat stages I, II, and III, respectively with a high statistically significant difference (*P* < 0.001) (Table 2)

**Table 2:** Relation between MDA and Sarnat scores shows that the mean MDA levels were 2.16 ± 0.31 μmol/l, 3.02 ± 0.67† μmol/l, and 4.13 ± 0.66†‡ μmol/l regarding Sarnat stages I, II, and III, respectively, in hypoxic neonates with highly statistical significant difference (*P* < 0.001**)**

Variable	Sarnat stage I	Sarnat stage II	Sarnat stage III	F	df	*P* value¶
MDA(nmol/ml)	2.16 ± 0.31	3.02 ± 0.67†	4.13 ± 0.66†‡	39.491	2, 39	**< 0.001**

Data are mean ± SD

*F* F statistic, *df* Degree of freedom

¶One-way analysis of variance (ANOVA)

†*P* value < 0.05 vs. Sarnat stage I (Tukey-Kramer test)

‡*P* value < 0.05 vs. Sarnat stage II (Tukey-Kramer test)

**Fig. 4:**
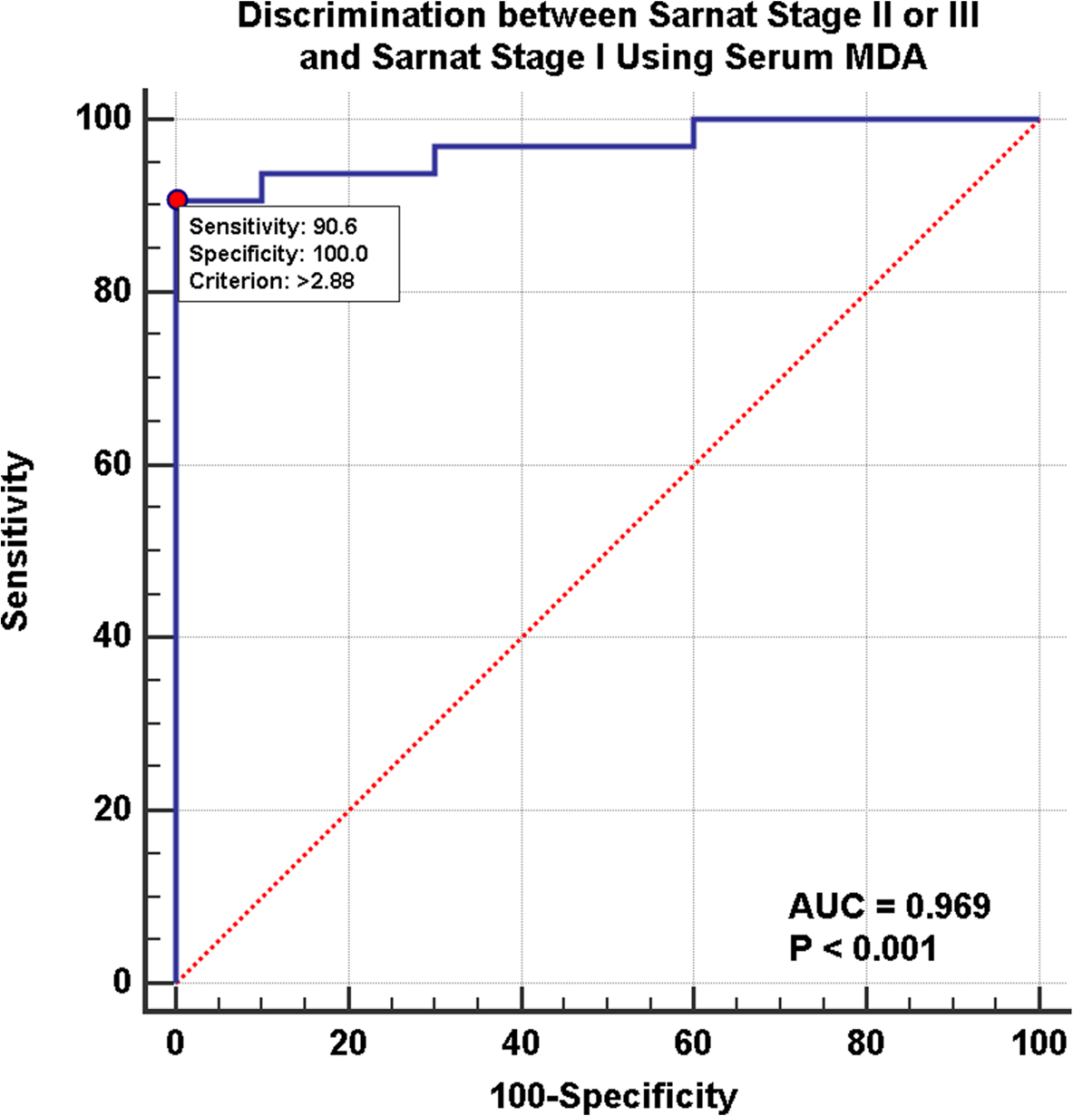
ROC curve analysis for discrimination between Sarnat stage II or III and Sarnat stage I patients using serum MDA levels. Cut-off MDA level is > 2.88 nmol/ml and area under the curve is 0.969. Sensitivity of MDA in Sarnat II or III compared to stage I was 90.62% and sensitivity was 100%

**Fig. 5:**
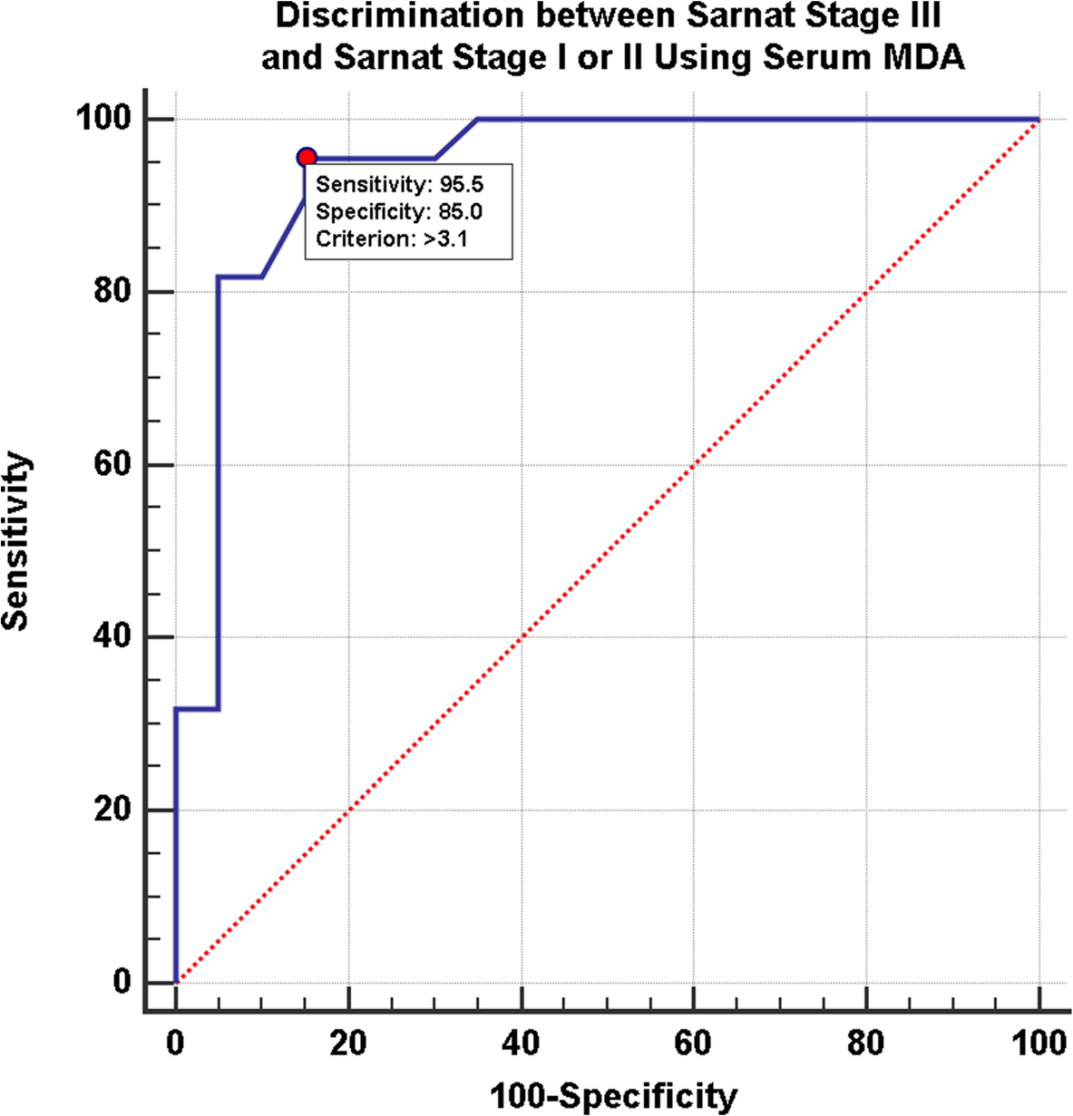
ROC curve for discrimination between Sarnat stage III and Sarnat stage I or II patients using serum MDA levels. Area under the curve was 0.942. Cut-off MDA levels was > 3.1 nmol/ml. Sensitivity of MDA in Sarnat stage III compared to stages II or I was 95.45% and specificity was 85%

ROC curves demonstrated a cut-off MDA level of > 2.88 nmol/ml between Sarnat stage I and Sarnat stage II/III in hypoxic patients; area under the curve was 0.969 with a 90% sensitivity and 100% specificity (Fig. 4). As for discrimination between Sarnat stage III and Sarnat stage I/II, cut-off level for MDA was > 3.1 nmol/ml; area under the curve was 0.942 with a 96% sensitivity and 85% specificity (Fig. 5). Finally, MDA levels correlated with outcome as well as severity; those with MDA levels > 2.88 nmol/ml had a longer hospital stay and were less likely to survive (Table 3).

**Table 3:** Relation between MDA and survival

Variable	Survivors (*n* = 24)	Non-survivors (*n* = 18)	t	df	*P* value¶
Serum MDA(nmol/ml)	2.94 ± 0.96	4.00 ± 0.76	− 3.864	40	**< 0.001**

Data are mean ± SD

*t* T statistic, *df* Degree of freedom

Unpaired *t* Test

**Fig. 6:**
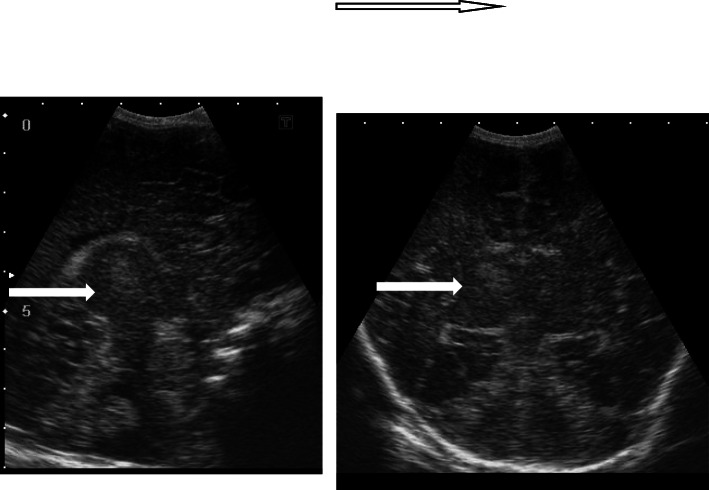
Sagittal and coronal US image with Sarnat stage II showing right thalamic and smaller left thalamic focal echogenic areas (white arrow), representing thalamic hypoxic insult lesions

**Fig. 7:**
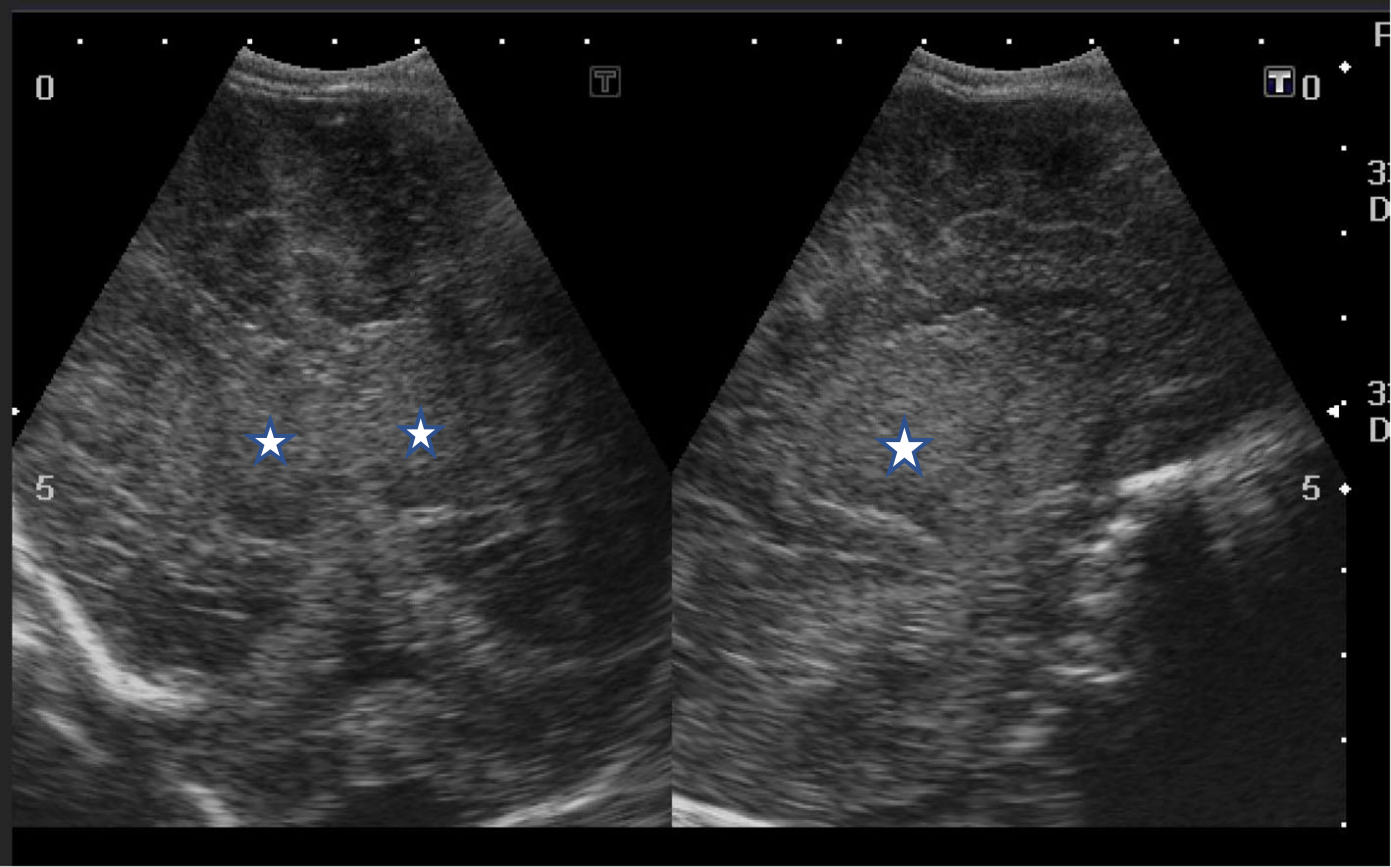
Coronal and sagittal US image of a neonate with Sarnat stage III showing severe hypoxic ischemic insult in terms of cerebral cortex and bilateral thalamic (white stars) and basal ganglia diffuse increased echogenicity with marked edema and compressed ventricles

**Fig. 8:**
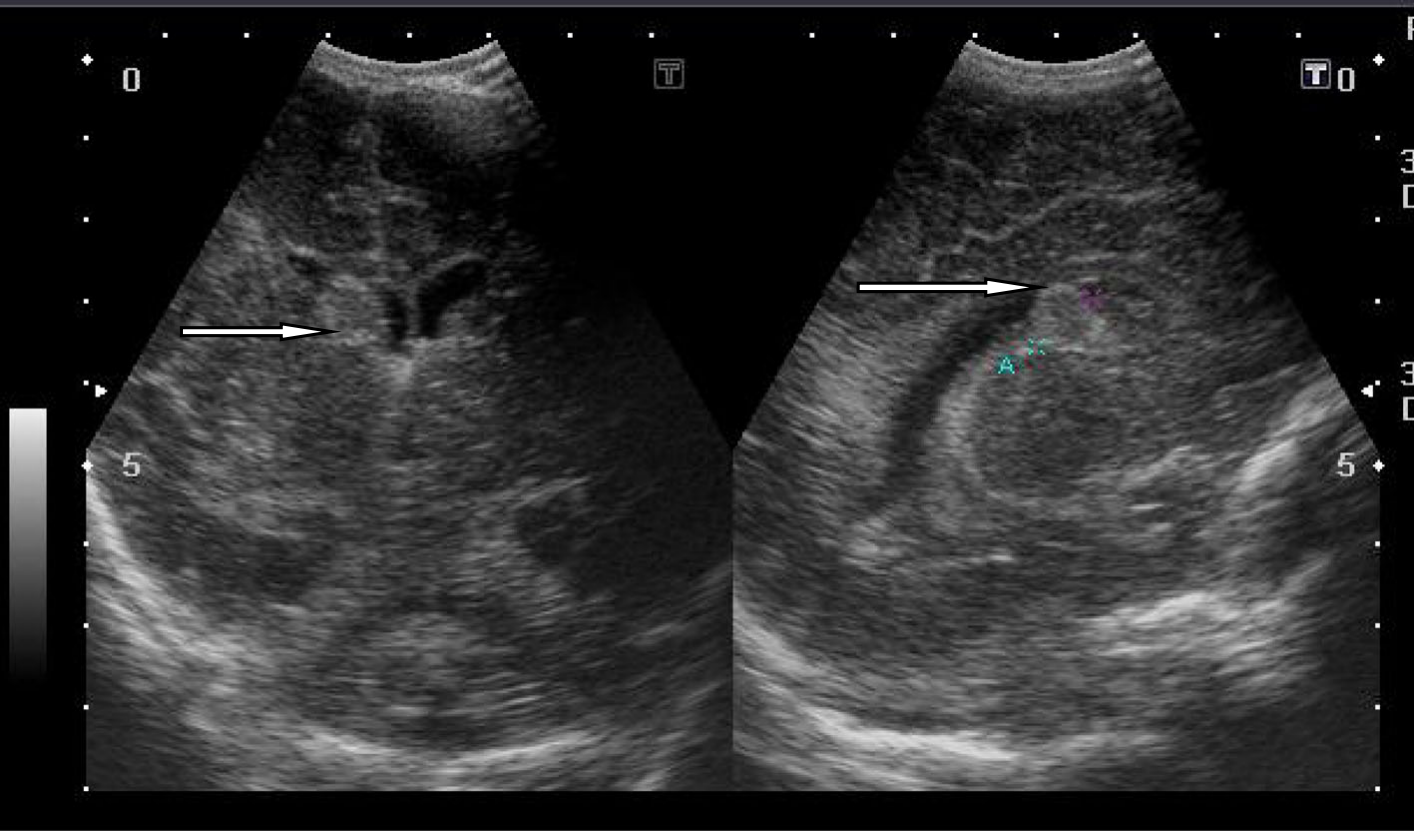
Coronal and sagittal US of a neonate with Sarnat stage II showing large echogenic subependymal hemorrhage of the right lateral ventricle, extending into the lumen representing grade I periventricular hemorrgae with impending intra ventricular extension

**Fig. 9:**
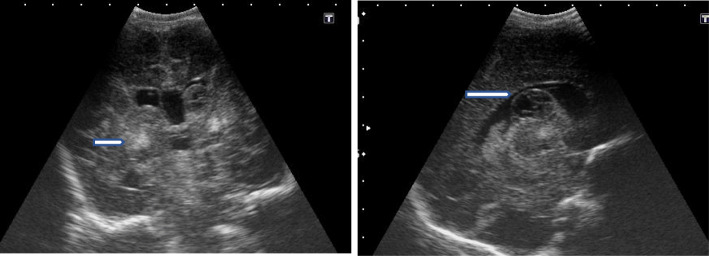
Coronal and sagittal US of a neonate full term. CS, cyanoses, Apgar scoring 2 and 4 at 1 and 5 min, anteparum hge, mechanical ventilator, and meconium aspiration syndrome. Sarnat stage III. Cranial US revealed aging subependymal hge (arrow), bilateral basal ganglia focal ecchognic areas (arrow head), and severe ischemia

In this study, cranial ultrasound was conducted for 37/42 hypoxic neonates and revealed 21/37 normal scans, while abnormal cranial US findings were found in 16 patients. We divided these 16 positive scans into ischemic pathology related to HIE (11/16) and non-ischemic (5/16). Ischemic cases included 4 with persistent diffuse subcortical hyperecchogenicities, 3 with unilateral thalamic echogenic foci, 2 with increased ecchogenicity and diffuse basal ganglia with brain edema, and 2 with focal subcortical white matter echogenic focus. Non-ischemic cranial ultrasound findings included 3 with obstructive hydrocephalus, and 2 with corpus callosum agenesis (Figs 6, 7, 8, and 9)

There was a significant difference between positive ischemic cranial ultrasound findings and Sarnat stages (*P* = 0.016). The majority of positive ischemic US findings were present in Sarnat stage 3 (63%) and Sarnat stage 2 (37%) (Table 4).

**Table 4:** Relation between US evidence of HIE and Sarnat stages

	HIE by US (*n* = 11)		No HIE by US (*n* = 21)				
Sarnat stage	N	%	N	%	χ^**2**^	df	***P*** value¶
Stage I	0	.0%	10	47.6%	5.782	1	**0.016**
Stage II	4	36.4%	4	19.0%			
Stage III	7	63.6%	7	33.3%			

χ^2^ chi-squared statistic, *df* Degree of freedom

Chi-squared test for trend

**Fig. 10:**
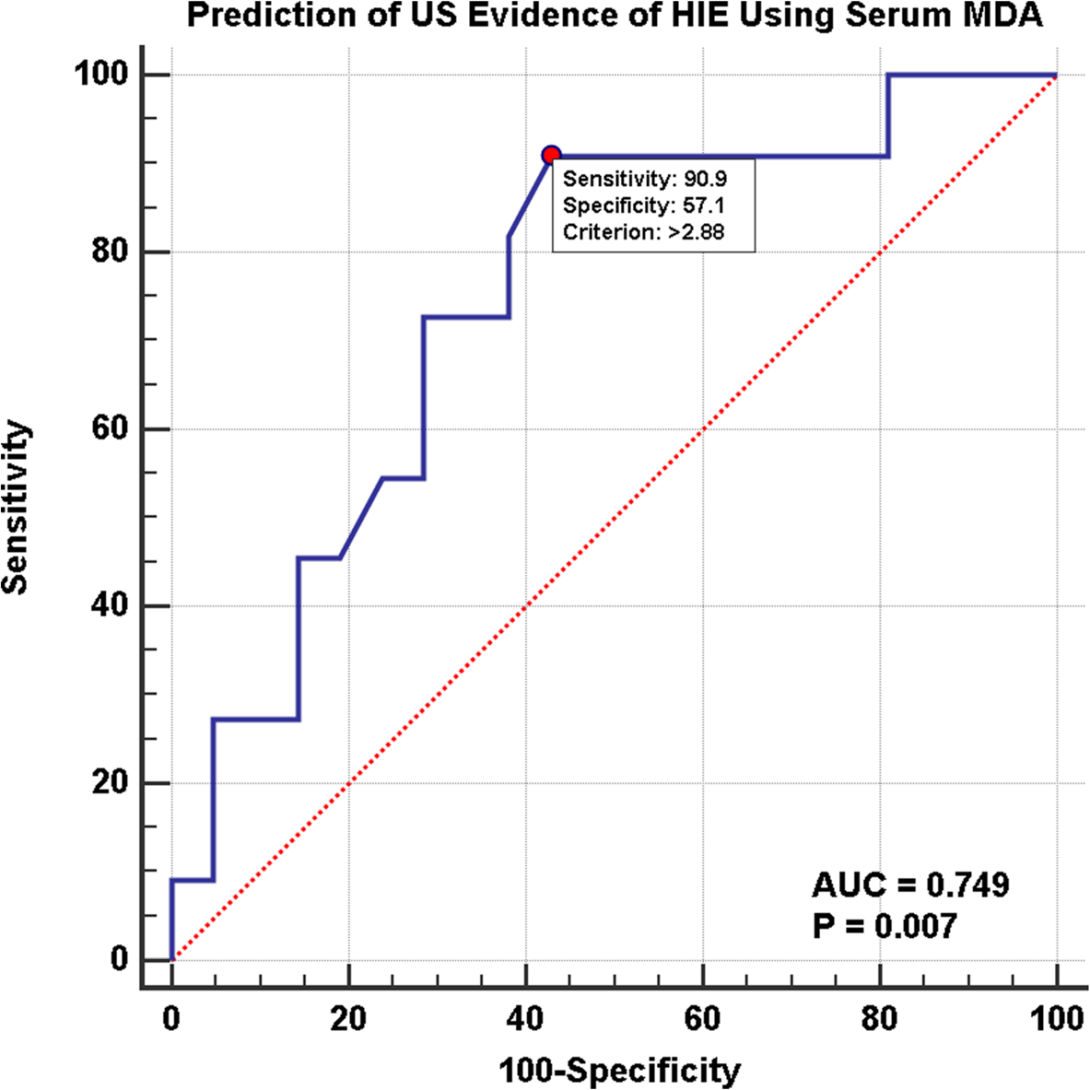
ROC curve for prediction of cranial US evidence of HIE using serum MDA. There is a highly significant difference between positive and negative US findings using MDA levels (*P* = 0.007). Area under curve was 0.749. Cut-off MDA in hypoxic neonates with positive US findings was > 2.88 nmol/ml. Sensitivity of MDA in positive US findings was 90.9% and specificity was 57.14%

As for correlation of HIE babies and cranial ultrasound findings, MDA levels were significantly higher in hypoxic neonates with positive ischemic US findings compared to those with negative US findings (*P* value = 0.018) (Table 5). Cut-off MDA level in hypoxic neonates with positive ischemic US findings was > 2.88 nmol/ml. Sensitivity of MDA in positive ischemic US findings was 90.9% and specificity was 57.14% (Fig. 10).

**Table 5:** Relation between MDA and presence of cranial ultrasound evidence of HIE

Variable	+ve findings by US (*n* = 11)	−ve findings by US (*n* = 21)	T	df	*P* value¶
Serum MDA (nmol/ml)	3.64 ± 0.83	2.84 ± 0.86	2.511	30	**0.018**

Data are mean ± SD

*t* T statistic, *df* Degree of freedom

¶Unpaired *t* Test

## Discussion

Hypoxic ischemic encephalopathy (HIE) remains a problem of great concern worldwide especially in developing countries. The occurrence of a neurological syndrome can be an indicator of an insult to the brain. HIE is an important cause of perinatal mortality and lifelong neurodevelopmental morbidity [11].

This was a prospective study conducted on 84 neonates in the period between September 2014 and August 2016. The study included 42 newborns fulfilling the criteria of hypoxia ischemia as a patient group and 42 normal newborns as a control group.

In terms of mode of delivery, studies have shown disparity regarding its relation to HIE [12, 13]. The timing of HIE may have a role in the variability of study results, whether the HIE was originally antepartum or whether it occurred as part of a difficult or complicated delivery; which may be a reason for varying conclusions on this point. Our study similar to another study by Alireza and Shirin [14] showed no statistical significance between the two groups in terms of mode of delivery.

Concerning hypoxic ischemic perinatal events, we found a highly significant difference regarding Apgar scores at 1 and 5 min compared to the control group (*P* < 0.00). These results stand in accordance with studies done by Nicole et al. and Florio et al. [15, 16], who reported that Apgar scores at 1 and 5 min were significantly lower in hypoxic than control newborns. In another study, Dede [17] and his colleagues mentioned that there was a significant difference between hypoxic and control groups regarding Apgar score but only at 1 min. Apgar score still remains an important tool in newborn assessment and monitoring the progress of resuscitation in a resource-poor setting. Both the 1st and 5th min Apgar scores showed a good correlation with clinical features of severe forms of HIE [18].

Median PH for the hypoxic group in this study was 7.14 and median base excess was − 14 mmol/L. Other studies have also found that profound metabolic alterations were present in hypoxic babies as compared to controls, whether in pH [3] and/or BE [19]. This was explained by Shah et al. [20] by demonstrating that there was a rise in organ injury rate as the arterial pH values decreased and base excess value dropped due to the fact that hypoxia causes anaerobic metabolism with high incidence of metabolic acidosis resulting in organ damage.

Hypoxia and ischemia can cause damage to almost every tissue and organ of the body and various target organs are involved. Gupta et al. [21] have reported injury to the kidneys in 50%, followed by central nervous system, cardiovascular system, and lungs. The most common system affection in our study was renal dysfunction (99%) followed by metabolic acidosis (91%) then pulmonary hypertension (60%). This is in agreement with another Egyptian study done by Mohammed et al. [22] who revealed that renal dysfunction was the most common followed by pulmonary dysfunction.

Since malondialdehyde (MDA) is one of the reactive metabolic products resulting from the effect of free oxygen radicals on tissues and from a series of reactions during lipid peroxidation [2], it can therefore, be used as a predictor for determining the presence and severity of HIE [3]. In our study, serum MDA levels were highly significant in hypoxic neonates (3.40 ± 1.02) nm/ml compared to the control group (0.77 ± 0.17) nm/ml [*P* < 0.001). This is in agreement with many other studies that showed MDA levels in hypoxic babies were highly significant compared to the control groups [23–25], indicating an association between perinatal asphyxia and oxidative stress.

In this study, there was a highly significant positive correlation between MDA and grading of encephalopathy; MDA levels were significantly higher in Sarnat stages II and III compared to Sarnat stage I (*P* < 0.001) as was mentioned by Kirimi et al. [24]. We also found a statistically significant positive correlation between rising MDA levels and progressive severity of encephalopathy as supported by Banupriya et al. [26]. Concerning neurological symptoms, as is mentioned in other studies [27], we similarly found serum MDA to be significantly higher in the patients who developed seizures compared to those who did not, highlighting the increasing severity of neurological symptoms with rising MDA levels.

Cranial US can be used to detect hemorrhage, periventricular leukomalacia (PVL), edema, and hydrocephalus [28]. The most common head sonography findings in neonates with hypoxic-ischemic injury are brain edema with echogenic subcortical white matter [29], which is consistent with finding by Van Wezel-Meijler [30], as well as findings in our study. Another study found intracranial hemorrhage and diffuse cerebral edema to be the most common ultrasound finding in HIE [31].

We found that MDA levels were significantly higher in hypoxic neonates with positive ischemic US findings compared to those with negative US findings with a cut-off MDA value of > 2.88 nmol/ml. We found a significant difference between positive ischemic cranial ultrasound finding and Sarnat stages; the majority of ischemic US findings were present in Sarnat stages II and III, which reveals a significant correlation between MDA, neurological deficits, and radiological findings of hypoxic neonates.

A cut-off level is essential to create a marker for clinicians to use for diagnosis and management of their patients. The significant MDA cut-off level that reappeared in our study was 2.88 nmol/ml. This number was significant for discrimination of Sarnat stages higher than I, earlier onset of seizures, positive cranial ultrasound hypoxic ischemic findings, longer hospital stay, and higher chances of mortality.

One of the limitations of this study was the inability to perform brain MRIs for these patients since our facility does not have a portable machine, and transporting these critically ill babies was not feasible. A larger study with a bigger sample population would perhaps have given more weight to our work but considering the scarcity of the condition, time factor was a restriction.

## Conclusion

Serum MDA sensitivity and specificity was 100% in hypoxic neonates which makes it a reliable diagnostic marker of oxidative stress in perinatal asphyxia. The significant correlation between Sarnat stages and serum MDA levels also provides us with a prognostic marker for cerebral damage and neurological outcome for neonates with HIE. An important cut-off value for MDA was > 2.88 nmol/ml which differentiated higher Sarnat stages, earlier onset of seizures, positive cranial ultrasound hypoxic findings, longer hospital stays, and higher chances of mortality.

Even though the sensitivity and specificity of cranial ultrasound for newborns with HIE is weak, it is still considered a noninvasive, relatively low-cost screening tool of the hemodynamically unstable neonate. Ultrasound was found to have low sensitivity in the diagnosis of HIE especially in milder grades. However, combination of ultrasound with measuring MDH concentration could act as diagnostic marker and a predictor of disease severity"

## Data Availability

Not applicable

## Abbreviations

HIE: Hypoxic ischemic encephalopathy
MDH: Malondialdehyde
NICU: Neonatal intensive care unit
US: Ultrasound
PVL: Periventricular leukomalacia
GA: Gestational age
